# Sclerosing Haemangiomas of the Liver: Two Cases of Mistaken Identity

**DOI:** 10.1155/2009/473591

**Published:** 2009-12-29

**Authors:** C. Lauder, G. Garcea, H. Kanhere, G. J. Maddern

**Affiliations:** Department of Hepatobiliary and Upper Gastrointestinal Surgery, The Queen Elizabeth Hospital, 28 Woodville Road, Adelaide, SA 5011, Australia

## Abstract

We describe two cases where patients undergoing hepatic resection for metastatic disease of colorectal origin were found to have concomitant sclerosing haemangiomas. The typical radiological and histological appearances of these lesions are discussed.

## 1. Introduction

Haemangiomas constitute the largest group of noncystic liver lesions [1], as a result they frequently present as confounding lesions when staging and investigating intra-abdominal malignancies. The sclerosed variant of liver haemangiomas is considerably less common. This describes two patients with metastatic colorectal malignancy in whom sclerosed haemangiomas were also identified.

## 2. Case 1

The first patient, a 72-year-old male, presented with large bowel obstruction secondary to a rectal cancer which was treated with a defunctioning colostomy and adjuvant chemoradiotherapy. Having completed the course of chemotherapy a staging CT was performed which demonstrated an ill-defined low density lesion in segment 4a. Dynamically this had enlarged from a CT scan 2 months previously. There was mild contrast enhancement of the lesion between arterial and venous phases with an increase of approx 5HU in density between the two phases. Another lesion was noted in segment 7, again low density, but stable in size when compared to the previous study. This showed less enhancement when compared to the lesion in 4a. Both these lesions were thought to be consistent with metastatic deposits. 

The patient underwent a routine ultra-low anterior resection with covering loop ileostomy. It was noted at the time of the laparotomy that there was a palpable metastasis adjacent to the falciform ligament. Further imaging for planning of the liver resection showed both lesions to have enlarged in size although the lesion in segment VII showed a smaller increase and was indeterminate in nature. At operation two surface liver lesions were seen and wedge excision was performed. The histology revealed a metastatic deposit of adenocarcinoma within segment 4a whilst segment VII showed a sclerosed haemangioma.

## 3. Case 2

The second patient, an 84-year-old male, had previously undergone a right hemicolectomy for adenocarcinoma and was noted at the time of the resection to have hepatic metastases. Having received chemotherapy postoperatively he was restaged to assess the feasibility of hepatic resection. The CT demonstrated four lesions in total, with sub centimetre hypodense lesions in segments 2 and 5, and larger lesions in segments 4b and 6 (Figure 2). The two larger lesions were thought to be consistent with malignant disease whilst the smaller segment 2 lesion was indeterminate and the abnormality in segment 5 was in keeping with a simple cyst. At laparotomy the lesions were further evaluated with intraoperative ultrasound. This confirmed the lesions in segments 2 and 5 to be cystic in nature and the patient therefore proceeded to a partial right hepatectomy without incident. Upon histological sectioning of the specimen only one metastatic deposit was found in segment 6, with the segment 4b lesion being a sclerosing haemangioma.

## 4. Discussion

These cases highlight the difficulty in differentiating between benign and malignant diseases in staging colorectal hepatic metastases. Furthermore we demonstrate the occurrence of rare hepatic lesions in mimicking malignant disease. Some authorities consider haemangiomas to be benign congenital hamartomas. They are composed of masses of blood vessels that are atypical or irregular in arrangement and size. Aetiology remains unknown with no definite familial or genetic mode of inheritance being described. When fibrosis occurs in an existing haemangioma then it may progress to the sclerosed variant. It is difficult to ascertain the precipitating event for this to occur. There are numerous reports of haemangiomas rupturing spontaneously or in association with trauma. It would therefore seem plausible that minor haemorrhage and thrombosis within a haemangioma may instigate fibrotic progression to a sclerosed haemangioma. Makhlouf et al. suggested that mast cells play a pivotal role in the development of a sclerosed haemangioma, perhaps representing a distinct histological subtype of liver lesion [2]. 

Not all haemangiomas remain stable in size or display typical appearances on imaging (due to the development of fibrous change) and this may lead to difficulty in accurately predicting the true nature of these incidental lesions when they occur. CT features suggestive of sclerosed haemangiomas include geographic outline, capsular retraction, decrease in size over time, and loss of previously seen regions of enhancement. Additional features include the presence of transient hepatic attenuation difference, rim enhancement, and nodular regions of intense enhancement as seen in typical haemangiomas [3]. Alternate imaging modalities such as MRI should be considered for further evaluation of indeterminate lesions. Radiological appearances must also be correlated to the treatment regimen specific to the patient. Adjuvant chemotherapy may lead to the change of a lesion on serial imaging, adding further weight to a diagnosis of malignancy.

**Figure 1:**
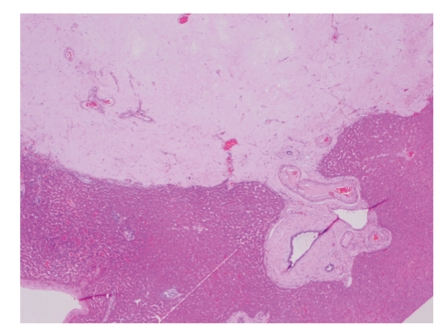
Sclerosed haemangioma with feeding vessel.

**Figure 2:**
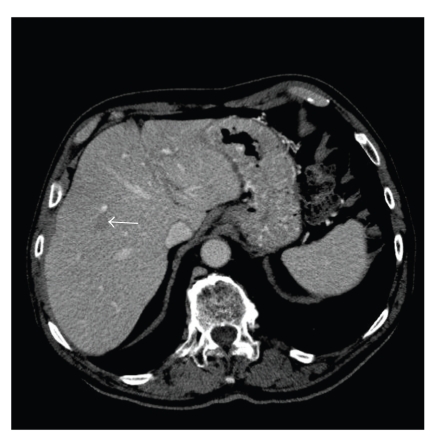
CT Scan showing segment 4b sclerosing haemangioma.

The common histological appearances of sclerosed haemangiomas consist of multiple thin walled vessels within a hypocellular stroma demonstrating varying degrees of fibrosis and sclerosis (Figure 1). Macroscopically a haemangioma is usually reddish-blue and well demarcated from surrounding tissue. However the sclerosed variant is seen as a pale nodule if there is significant fibrosis present. The cell type of origin is mesenchymal and as such the lesions can occur almost anywhere [4].

Whilst the prevalence of haemangioma has been reported as high as 20% when found incidentally at autopsy [5], the sclerosed variant is considered much rarer. Sclerosis may occur at varying degrees to mask the appearance of the haemangioma. When complete the lesion takes on a much more fibrotic appearance exhibiting few of the characteristics of the lesion from which it originated. In patients whom have metastatic liver lesions any indeterminate lesion must be treated with extreme caution to ensure that there is no residual disease. 

One of the key questions in the management of patients in this scenario is whether to biopsy the lesion in question at the time of the operation or to proceed straight to formal resection. Whilst thorough preoperative investigation will guide the surgeon in their management, this decision can only be made at the time of operation. We would certainly not condone preoperative biopsy of any liver lesions in the context of planning definitive treatment for suspected colorectal hepatic metastases. However, it is conceivable that if excision of an indeterminate lesion may significantly increase the length or risk of surgery (especially in high-risk patients) then an intraoperative biopsy with frozen histology could be considered. Patients should always be informed of the potential for benign pathology when surgery is recommended for indeterminate lesions. Hepatic resection for benign conditions is only indicated where there are persistent symptoms, uncertainty regarding the diagnosis or the risk of malignant transformation [6]. However a systematic review failed to demonstrate any significant evidence to support the elective resection of benign liver tumours, although the number of patients was limited and no randomised control trial data is available [7].

Cross-sectional imaging, whilst continually improving in detail, can still not always differentiate between certain benign lesions and malignant disease. This is particularly true for small lesions less than 5 mm. In the context of multiple mixed lesions tumour makers will not help quantify or localise metastatic deposits. Whilst a PET scan may help distinguish between benign and malignant disease there are still instances where benign pathologies can mimic malignancy.

## 5. Conclusion

In both cases the clinical index of suspicion for metastatic spread was high given the advanced nature of the colorectal cancers. Despite accurate preoperative investigation it may not always be possible to delineate between a benign lesion such as a sclerosing haemangioma and a true metastatic deposit, especially when the two coexist. The typical imaging features of sclerosed haemangioma have recently been published as discussed earlier. However no imaging technique can be 100% accurate. Tumour markers will not differentiate between the two pathologies in this scenario whilst both false positives and negatives can occur with PET scans [8]. If a lesion is not readily resectable, an intraoperative biopsy can provide a tissue diagnosis to negate the risk of a major resection. When there is persistent doubt regarding the diagnosis it would seem prudent to plan for resection of the lesion with the patient prepared for the eventuality of benign pathology. Given the relatively common occurrence of haemangiomas combined with a high incidence of colorectal cancer, cases such as these may become a more common event.
